# Biomarkers of Venous Thromboembolism Recurrence after Discontinuation of Low Molecular Weight Heparin Treatment for Cancer-Associated Thrombosis (HISPALIS-Study)

**DOI:** 10.3390/cancers14112771

**Published:** 2022-06-02

**Authors:** Remedios Otero, Aurora Solier-López, Verónica Sánchez-López, Julia Oto, Elena Arellano, Samira Marín, Luis Jara-Palomares, Teresa Elías, María Isabel Asencio, Isabel Blasco-Esquivias, María Rodríguez de la Borbolla, José María Sánchez-Díaz, Macarena Real-Domínguez, Emilio García-Cabrera, Francisco Javier Rodríguez-Martorell, Pilar Medina

**Affiliations:** 1 Medical Surgical Unit of Respiratory Diseases, Hospital Universitario Virgen del Rocío, 41013 Seville, Spain; aurora.solier@salud.madrid.org (A.S.-L.); samira.marin.sspa@juntadeandalucia.es (S.M.); luisj.jara.sspa@juntadeandalucia.es (L.J.-P.); teresa.elias.sspa@juntadeandalucia.es (T.E.); isabel.asensio.cruz.sspa@juntadeandalucia.es (M.I.A.); jmaria.sanchez.diaz.sspa@juntadeandalucia.es (J.M.S.-D.); 2 Instituto de Biomedicina de Sevilla (IBiS), Consejo Superior de Investigaciones Cientificas (CSIC), Universidad de Sevilla, 41013 Sevilla, Spain; vsanchez-ibis@us.es (V.S.-L.); marellano-ibis@us.es (E.A.); 3 Centro de Investigación Biomédica en Red de Enfermedades Respiratorias (CIBERES), Instituto de Salud Carlos III, 28029 Madrid, Spain; 4 Haemostasis, Thrombosis, Arteriosclerosis and Vascular Biology Research Group, Medical Research Institute Hospital La Fe (IIS La Fe), La Fe University and Polytechnic Hospital, 46026 Valencia, Spain; julia_oto@iislafe.es (J.O.); medina_pil@gva.es (P.M.); 5 Internal Medicine, Emergency Service, Virgen Macarena Hospital, 41009 Seville, Spain; mariai.blasco.sspa@juntadeandalucia.es; 6 Medical Oncology, Virgen de Valme Hospital, 41014 Seville, Spain; maria.rodriguezborb.sspa@juntadeandalucia.es; 7 Departamento de Medicina Preventiva y Salud Pública, Universidad de Málaga, 29071 Málaga, Spain; macarenareal@uma.es; 8 Departamento de Medicina Preventiva y Salud Pública, Universidad de Sevilla, 41009 Seville, Spain; egcabrera@us.es; 9 Department of Hematology, Hospital Universitario Virgen del Rocio, 41013 Seville, Spain; fjavie.rodriguez.martorell.sspa@juntadeandalucia.es

**Keywords:** biomarkers, venous thromboembolism, recurrence, cancer, cancer-associated thrombosis

## Abstract

**Simple Summary:**

The duration of anticoagulant treatment for venous thromboembolism in cancer patients is not well defined. The decision to suspend or re-introduce anticoagulation is based on an individual assessment between thrombotic and hemorrhagic risk. In this study, three biomarkers have shown the ability to help the clinician in decision making. Although these results should be evaluated in further studies, they open up a possibility of change in the management of cancer associated thrombosis patients.

**Abstract:**

The most appropriate duration of anticoagulant treatment for cancer-associated venous thromboembolism (CAT) remains unclear. We have conducted a prospective multicenter study in CAT patients with more than 6 months of anticoagulant treatment to predict the risk of venous thromboembolism (VTE) recurrence after anticoagulation discontinuation. Blood samples were obtained when patients stopped the anticoagulation, at 21 days and at 90 days. In each sample we assessed different coagulation-related biomarkers: D-dimer (DD), high-sensitivity C-reactive protein (hs-CRP), P-selectin (PS), phospholipids, soluble tissue factor, factor VIII and the thrombin generation test. It was evaluated 325 CAT patients and 166 patients were included in the study, mean age 64 ± 17 years. VTE recurrence until 6 months after stopping anticoagulation treatment was 9.87% [95% confidence interval (CI): 6–15]. The biomarkers sub-distribution hazard ratios were 6.32 for ratio DD basal/DD 21 days > 2 (95% CI: 1.82–21.90), 6.36 for hs-CRP > 4.5 (95% CI: 1.73–23.40) and 5.58 for PS > 40 (95% CI: 1.46–21.30) after 21 days of stopping anticoagulation. This is the first study that has identified the DD ratio, hs-CRP and PS as potential biomarkers of VTE recurrence in cancer patients after the discontinuation of anticoagulation treatment. A risk-adapted strategy may allow the identification of the optimal time to withdraw the anticoagulation in each CAT patient.

## 1. Introduction

Cancer occurrence in conjunction with its treatment and the intrinsic characteristics of the patients increases the risk of developing a thromboembolic disease. Indeed, the overall risk of venous thromboembolism (VTE) is increased 7-fold in oncologic patients compared with the general population [1]. Patients with cancer-associated thrombosis (CAT) have a 3-fold shorter one-year survival rate compared to patients with cancer without VTE [2]. The risk factors for VTE in cancer patients can be divided into individual patient risk factors (age, race, comorbidities, immobility, previous history of VTE), risk factors related to cancer (histology, stage, localization of neoplasm, time since diagnosis) and risk factors related to treatment (surgery and hospitalization, chemotherapy, angiogenesis inhibitors, central venous catheters) [3]. The most thrombogenic tumor types are pancreas, head/neck, central nervous system, upper gastrointestinal, endocrine, lung and colorectal [4], with the greatest VTE incidence occurring within the first year of follow-up in metastatic cancer [5].

The hazard of death is increased 3-fold among CAT patients with recurrent VTE [6], revealing that the prevention of VTE recurrence may be of paramount importance for long-term survival in these patients. Conversely, cancer patients also have a high risk of anticoagulant-associated major bleeding [6]. Consequently, the optimal dose and duration of anticoagulation in CAT patients remains unclear.

Several clinical assessment scores have been proposed for thrombotic risk stratification in cancer patients [7,8,9,10,11]. These scores comprehend tumor-related factors, cancer subtype and baseline laboratory values. The most well-known score is the Khorana score and The Vienna Cancer and Thrombosis (CATS) Study proposes an expanded risk Khorana model incorporating two additional biomarkers, soluble P-selectin (sP-selectin) and D-dimer (DD), considerably improving the VTE risk prediction [12]. As for all clinical assessment scores, several limitations have been raised [13,14,15] and current tools for predicting and monitoring the risk of VTE are inadequate. Nonetheless, these risk assessment scores are ineffectual in cancer patients with a previous VTE event, where clinical decisions regarding anticoagulant therapy duration ought to be based on the estimation of the risk of VTE recurrences of each individual patient. Guidelines establish a duration of anticoagulant treatment in CAT patients up to 6 months. The anticoagulation beyond 6 months often depends on a case-by-case basis.

The incidence of VTE recurrence in CAT patients varies among studies, typically ranging from 10% to 20% [16]. The only available tool to specifically predict VTE recurrence in CAT patients is the Ottawa score [17]. This score, in its original or the modified version, classifies CAT patients as high or low risk to developing a VTE recurrence during the first 6 months of anticoagulation treatment, and comprehends the following variables: sex, tumor type, previous history of VTE and tumor stage. However, no tools are available to monitor the risk of VTE recurrences in CAT patients after anticoagulation withdrawal.

Thus, the aim of our study was to identify a profile of clinical variables and coagulation-related parameters capable of predicting the risk of VTE recurrences in CAT patients after anticoagulant withdrawal.

## 2. Materials and Methods

### 2.1. Design

Longitudinal prospective multicenter study to detect VTE recurrences after withdrawal of anticoagulation in CAT patients. Approval was obtained from the Andalusian Bioethics Committee and all participants provided their written informed consent (clinicaltrials.gov identifier: NCT03134820).

### 2.2. Patients

Patients were recruited in three university hospitals of Spanish Health Service at Seville (Hospital Virgen Macarena, Hospital de Valme, and Hospital Virgen del Rocío) between December 2013 and September 2016. All patients provided written informed consent before enrolment.

Eligible patients were considered any active cancer patients (excluding nonmelanoma skin cancer) with objectively diagnosed pulmonary embolism (PE) and/or deep vein thrombosis (DVT) and treated using low-molecular-weight heparin (LMWH) for ≥6 months after the diagnosis. The exclusion criteria were: (1) a life expectancy of <6 months, (2) cancer in progression, (3) pregnancy, (4) residual vein thrombosis in DVT (defined as <0.3 cm of vein transversal diameter when a compression is exerted with the ultrasound transducer), (5) lupus anticoagulant or anticardiolipin antibodies (required two tests separated by 12 weeks), (6) no medical reason to continue treatment and (7) suspicion of chronic thromboembolic pulmonary hypertension (CTEPH), which was ruled out by the following questions: (a) Have you presented with dyspnea after PE event? (b) Have you presented with palpitations, chest tightness or fading without the known justification? (c) Have you had signs of congestive heart failure without the known etiology or justification (jugular engorgement, peripheral oedema, or ascites)?

### 2.3. Study Protocol

**Figure 1:**
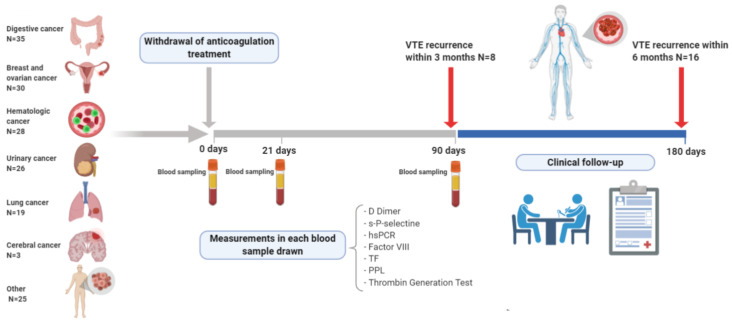
Plan of the HISPALIS Study.

The study plan is shown in Figure 1. Patients were treated with LMWH at a weight-adjusted dosage. After at least 6 months of anticoagulation treatment, the clinician proposed the enrollment to those patients with no exclusion criteria. Once anticoagulation was stopped, blood samples were obtained from all patients to test biomarkers on the day of anticoagulation withdrawal (baseline), 21 and 90 days later. All blood samples were analyzed in the same laboratory (Instituto de Biomedicina de Sevilla) using identical procedures. Patients were informed to detect symptoms of VTE recurrence, and a telephone number was provided to contact the clinicians to consult any suggestive symptoms of VTE recurrence. All patients were also followed after 180 days. Episodes of VTE recurrence, primary outcome, bleeding and all causes of death were recorded for analysis. When a patient suffers a recurrence of VTE, it will mean the end of follow-up for the patient, anticoagulant treatment will be started again and subsequent biomarker determinations that the patient has scheduled will be suspended.

### 2.4. Blood Samples and Biomarker Testing

Venous blood was collected with a 21-gauge needle (discarding the first 3 mL) using 3.5 mL of 9NC coagulation 3.2% sodium citrate Vacuette^®^ tubes (Greiner Bio-One, NC, USA). Citrated blood samples were centrifuged at 1500× *g* for 30 min at 4 °C within one hour of venipuncture. Plasma aliquots were stored at −80 °C until further use. Biomarkers were performed according to manufacturer’s instructions: high-sensitivity C-reactive protein (hs-CRP) (N High Sensitivity CRP, Dade Behring^®^; normal range: <5 mg/L) on a Behring Nephelometer II System; DD (Innovance^®^ D-Dimer Siemens Medical Solutions Diagnostics, Deerfield, IL, USA, lower limit of detection 0.099 mg/L fibrinogen equivalent units (FEU)); sP-Selectin (Human sP-selectin/CD62P Immunoassay, sensitivity: 0.121 ng/mL, specificity: recombinant and human P-selectin, R&D Systems, Minneapolis, MN, USA); and tissue factor (TF) (Imubind^®^ human Tissue Factor ELISA, sensitivity: 10 pg/mL approximately, specificity: TF-apo, TF and TF-VII complex and is designed such as there is no interference from other coagulation factors or inhibitors of procoagulant activity, American Diagnostica, Stanford, CT, USA). Factor (F) VIII activity was measured by coagulometric method on a Sysmex CA 7000 analyzer using FVIII-deficient plasma and APTT reagents (all from Siemens Healthcare Diagnostics, Newark, DE, EEUU). All assays were performed according to the manufacturer’s specifications and standard laboratory methods. The phospholipid procoagulant activity (STA-Procoag-PPL, Diagnostica Stago, Asnieres, France) assay was used to evaluate the PPL activity. Results are expressed as coagulation time (s), the shorter the coagulation time, the higher the PPL activity.

The thrombin generation test (TGT) was performed using the Calibrated Automated Thrombogram at 37 °C in a Fluoroskan Ascent reader (Thermo Labsystems) with 390/460 filters. The TG curves and the different parameters were obtained using the software Thrombinoscope (Thrombinoscope BV). A typical TGT reaction consisted of 80 µL platelet-poor plasma, 20 µL PPP reagent (Thrombinoscope BV) that includes 5 pM TF and 4 µM phospholipids and 20 µL fluorogenic substrate FluCa-Kit (Thrombinoscope BV) in the presence of CaCl_2_. Each reaction was performed in duplicate. In parallel, an additional reaction containing 20 µL thrombin calibrator (Thrombinoscope BV) was also performed for normalization.

### 2.5. Study Variables

Data were collected at the time of the enrolment concerning the patients’ demographic characteristics, VTE risk factors, VTE diagnosis (DVT and/or PE), anticoagulation treatment and oncological treatment before inclusion. The biomarker results (at baseline, 21 or 90 days) were collected. The main outcome variable was VTE recurrences up to 6 months after stopping the anticoagulant treatment. All-cause mortality and VTE-related mortality were determined using objective methods or via clinical consensus among the oncologists and pulmonologist or internist. Recurrent PE was defined with at least one of the following findings: (1) a new intraluminal filling defect in the sub-segmental or more proximal branches that were detected during spiral computed tomography; (2) a new intraluminal filling defect, the extension of an existing defect or a new sudden occlusion of vessels with a diameter of >2.5mm that was detected during pulmonary angiography; (3) a new perfusion defect (≥75% of a segment) with a local normal ventilation result (high probability) on the ventilation/perfusion lung scan; or (4) inconclusive findings from computed tomography, pulmonary angiography or ventilation/perfusion lung scan but with compression ultrasonography or venography revealing new or extended DVT in the lower extremities. Recurrent DVT was defined as (1) abnormal findings from compression ultrasonography with previous normal compression, (2) a substantial increase in diameter (≥4 mm) of the thrombus during full compression for previously non-compressible areas or (3) extension of an intraluminal filling defect, a new intraluminal filling defect, or an extension of venous non-visualization in the presence of a sudden cutoff during venography.

### 2.6. Statistical Analysis and Sample Size

Continuous variables were reported as mean ± standard deviation (SD) or median and interquartile range (IQR) according to normal or non-normal distribution, respectively. Categorical variables were reported as number and frequency.

We evaluated the optimal levels of DD, hs-CRP and sP-selectin for predicting VTE recurrence using a receiver operating characteristic (ROC) curve, the area under the curve (AUC) was >0.5 based on the standard error obtained using DeLong’s method.

A multivariate logistic regression model evaluated the predictive values of these biomarkers. Competing risk regression analysis of time to VTE recurrence was performed for significant biomarkers (Fisher’s exact test, *p* < 0.05). Wald’s test was used to assess the effect of a variable within the competing risk regression model. All analyses were performed using the IBM SPSS software (version 20), EPIDAT software (version 4.1) and R software (version 3.0.1) with the “survival” and “cmprsk” packages.

We assumed a recurrence rate of 10% during the first 6 months after discontinuation of anticoagulation treatment. Based on a safety level of 95% (1 − α), statistical power of 90% and a 15% mortality rate, the minimum required sample size was 113 patients.

## 3. Results

**Figure 2:**
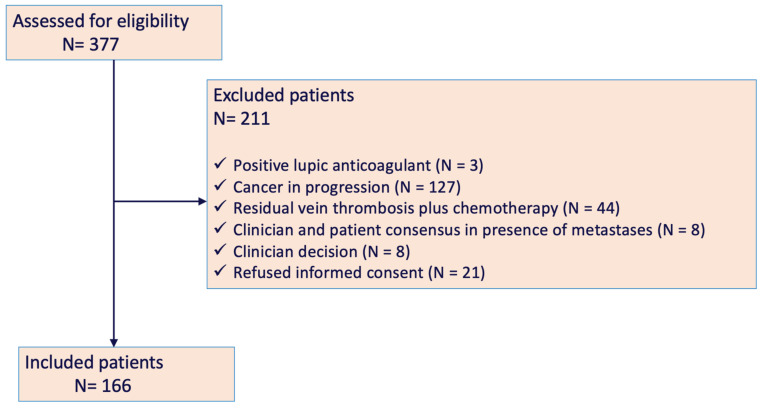
Flowchart of HISPALIS Study.

A total of 377 CAT patients, with LMWH beyond 6 months, were evaluated; finally, 166 patients were enrolled in the study. Figure 2 shows the flowchart of patients enrolled in the HISPALIS study. The clinical characteristics of the patients studied are summarized in Table 1, while the clinical characteristics of the excluded patients can be seen in Table S1. The mean age of the enrolled participants was 64 ± 17 years and 53.6% (N = 89) were men. There was a higher rate of recurrences in men (11/89; 12.4%) than in women (5/77; 6.5%) but this difference was not significant. Tumors were located at the following sites: gastrointestinal (N = 35, 21.1%), breast and ovarian (N = 30, 18.1%), hematologic (N = 28, 16.9%), urinary (N = 26, 15.6%), lung (N = 19, 11.4%), cerebral (N = 3, 1.8%) and other sites (N = 25, 15.1%). Nearly 40% of the patients had metastases. VTE presented as follows: 53.6% DVT, 28.3% PE, 11.4% PE plus DVT and 6.1% atypical VTE (N = 4 in inferior vena cava and iliac territory, N = 2 upper limb DVT and N = 1 splanchnic vein thrombosis).

The incidence of VTE recurrence within 3 months after anticoagulation withdrawal was 4.8%, six patients suffered DVT and two patients PE plus DVT. The VTE recurrence within 6 months was 9.6%, eleven patients suffered DVT and five patients PE plus DVT.

Of the 166 patients studied, six (3.61%) had bleedings before stopping the anticoagulant treatment, two of whom had major bleeding. The median ± IQR for the duration of anticoagulant treatment in all patients was 10 ± 11 months, with no differences between those with or without VTE recurrences (12 ± 14 vs. 10 ± 11 months; *p* = 0.102). No association was found between VTE recurrences and anticoagulation treatment for <12 months (6/61) vs. ≥12 months (4/53) (*p* = 0.75).

### 3.1. Incidence of VTE Recurrences after Anticoagulation Withdrawal

**Figure 3:**
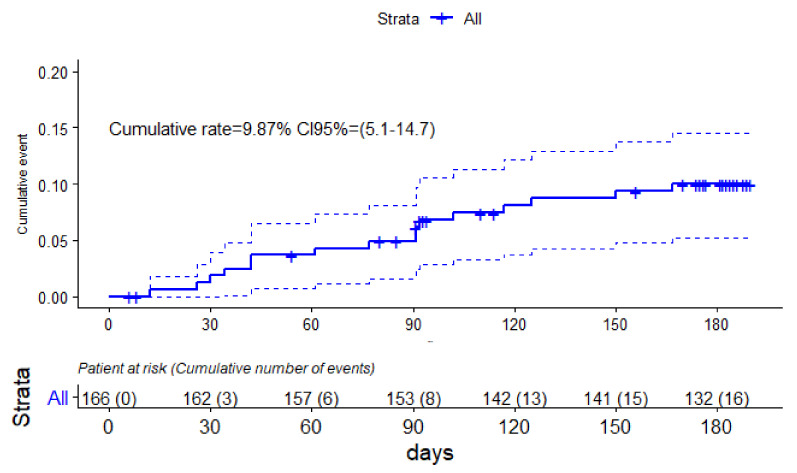
Kaplan–Meier curve of VTE recurrences in cancer patients after anticoagulation withdrawal.

In order to evaluate the existence of specific periods of higher VTE recurrence after anticoagulant withdrawal, we performed a Kaplan–Meier analysis. Figure 3 depicts the cumulative incidence of VTE recurrences for the follow-up time in our cohort of CAT patients.

Following 180 days of anticoagulant withdrawal, 8 VTE recurrences were registered and at the end of follow-up, 16 recurrences were registered. The cumulative rate of all follow-up period was 9.87% (95% CI: 5.1–14.7).

All recurrences were objectively diagnosed but anticoagulant treatment was restarted without objective diagnosis in nine patients (5.42%). The reason was an extremely high DD value according to the doctor’s interpretation that made a reintroduction of the anticoagulant treatment.

### 3.2. Evolution of Biomarkers after Anticoagulation Withdrawal

**Figure 4:**
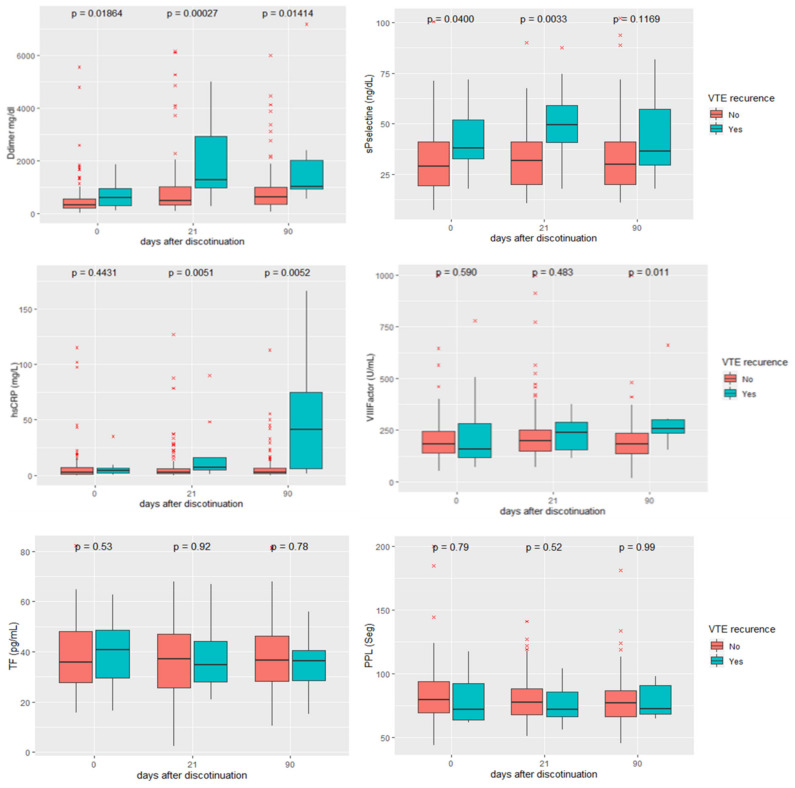
Longitudinal profile of coagulation-related parameters measured in CAT patients. D-dimer (DD), soluble P-selectin (sP-selectin), high-sensitivity C reactive protein (hs-CRP), factor VIII (FVIII), tissue factor (TF) and phospholipid procoagulant activity (PPL). Blue boxes refer to CAT patients with VTE recurrence and red boxes refer to patients without VTE recurrence. Parameters were measured at recruitment (the day of anticoagulation discontinuation), 21 days and 90 days after discontinuation of anticoagulation. Boxplots show median and interquartile range.

In each blood sample obtained from CAT patients (0 days, 21 days and 90 days after anticoagulation withdrawal) different parameters were measured and their levels were compared between patients with and without VTE recurrence. As seen in Figure 4, we found significantly increased DD levels at recruitment, at 21 days and 90 days after anticoagulation withdrawal. We also found a significant increase in sP-selectin at recruitment and at 21 days but not at 90 days after anticoagulation withdrawal. Additionally, we found higher levels of hs-CRP in those patients who suffered VTE recurrence 21 days and 90 days after anticoagulation withdrawal but not at recruitment. Finally, we found an increase in FVIII levels 90 days after anticoagulation withdrawal. No significant differences were evidenced in TF levels or PPL activity during follow-up.

**Figure 5:**
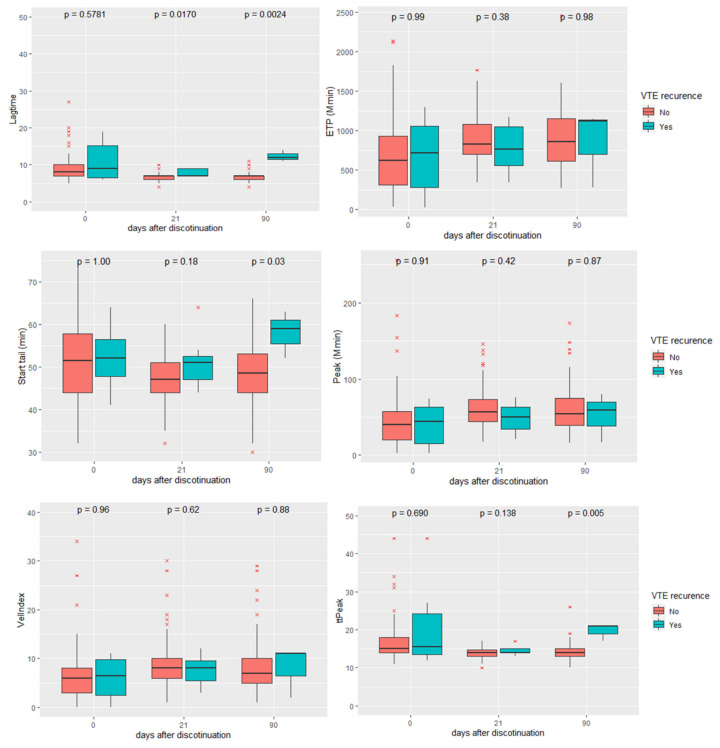
Longitudinal profile of the different parameters obtained from the thrombin generation test in CAT patients. Endogen thrombin potential (ETP), velocity index (Vel Index) and time to peak (ttPeak). Blue boxes refer to CAT patients with VTE recurrence and red boxes refer to patients without VTE recurrence. TGT parameters were measured at recruitment (the day of anticoagulation discontinuation), 21 days and 90 days after discontinuation of anticoagulation. Boxplots show median and interquartile range.

We also performed the TGT in each blood sample obtained and we compared the results of each TGT parameter between patients with and without VTE recurrence. We found a significant increase in the lagtime 21 days and 90 days after anticoagulant withdrawal. We also observed an increase in the ttPeak and start tail 90 days after anticoagulant withdrawal. No significant differences occurred in ETP, peak or VelIndex during follow-up (Figure 5).

### 3.3. Predictive Role of Biomarkers in Venous Thromboembolism Recurrence

To determine the predictive accuracy of thromboembolic recurrence biomarkers after withdrawal of anticoagulation, we constructed ROC curves considering the measurement performed at 21 days, according to the longitudinal profile of the analyzed parameters (see Figure 4. We also constructed ROC curves using the ratio between baseline and at 21 days for DD measurements. The 21-day DD/baseline DD ratio (DD ratio), hs-CRP and the sP-selectin value at 21 days rendered an AUC of 0.709, 0.71 and 0.747, respectively. This allowed us to determine different cut-off points: DD ratio > 2, hs-CRP > 4.5 mg/L and sP-selectin > 40 ng/dL.

The distribution of the hazard ratios (HR) of VTE recurrence in CAT patients without death competing risk and HR ratio of recurrence adjusted with competing risk (sHR) is presented in Table 2. The adjusted competing risks were 6.36 for hs-CRP > 4.5 (95% CI [1.73, 23.4], *p* = 0.005), 6.32 for DD ratio > 2 ([1.82, 21.90], *p* = 0.003), 5.58 for sP-selectin > 40 ([1.46, 21.30], *p* = 0.012) and 2.87 for male sex ([0.78, 10.5], *p* = 0.110) which was introduced in the model given the relevant role that sex had in previous studies concerning VTE recurrences [18].

Sensitive, specificity and predictive values of the selected biomarkers to estimate VTE recurrences in CAT patients and those of the modified Ottawa score are shown in Table 3. We observed that the three parameters, DD ratio > 2, sP-selectin > 40 ng/dL and hs-CRP > 4.5 mg/L, have a high predictive negative value and that all outperform the modified Ottawa score, which has a very low sensitivity. The best prediction of VTE recurrence was achieved when some of the biomarkers were above the cutoff point, reaching a sensitivity of 93.7% [67.7–99.6] and a PNV of 98.2% [89.2–9.9].

## 4. Discussion

Cancer-associated thrombosis is a common complication of the oncologic process thus anticoagulation is a common practice. LMWH has been the recommended standard of care for the treatment of CAT in numerous guidelines [19,20,21,22]. However, recent studies suggest the use of direct oral anticoagulants (DOACs) for the treatment of VTE in cancer patients, although LMWH is preferred when there is a risk of bleeding, the tumor originates in the digestive or urinary tract, there is a significant risk of interactions between DOAC and the drugs used to treat the patient, or when the thrombosis is related to central vein catheter/vascular port placement [23].

The optimal duration of therapy for CAT remains unclear as no randomized studies evaluating different therapy durations in cancer patients have been conducted. Nonetheless, the available data support the conclusion that patients with active cancer and particularly metastatic cancer undergoing therapy are at high risk for recurrent thromboembolism. Several scientific societies recommend at least 6 months of therapy [24,25,26]. Others recommends at least 3 months of therapy or for as long as cancer is active or patient undergoes treatment [27]. All the guidelines agree that the duration of therapy should be reassessed on a regular and dynamic basis according to the patient’s clinical situation, considering the risks and benefits of therapy and patient preferences. The reasons for stopping anticoagulation in our study are based on the exclusion criteria. Considering that this decision was based on a detailed case-by-case analysis, the results of the study may not be generalized, but the prospective nature of the design makes them at least useful for future studies based on our results.

All in all, in clinical practice the withdrawal of anticoagulation in cancer patients with a previous VTE event is a distressing clinical decision and the discovery of biomarkers to identify patients at high risk of VTE recurrences is of paramount importance. Presently, the Ottawa score, in its original and modified versions, is the only available tool to stratify the risk of VTE recurrence in CAT patients [17]. Both versions of the score include the clinical variables sex, tumor type, previous history of VTE and tumor stage. The original Ottawa score can reliably identify CAT patients at a high risk of recurrent events, whereas the modified score is best suitable for identifying CAT patients with a low risk of VTE recurrence. Nonetheless, this score could be improved by adding further coagulation-related parameters such as those proposed in the present study. Our results about the modified Ottawa score are consistent with the study of Y. Nishimoto et al. [28], which showed a modest discriminating power to predict the risk of recurrence with a C-statistic of 0.63 (95% CI, 0.55–0.71).

The value of DD as a marker of VTE recurrences in cancer patients has been controversial. In a prospective study of 117 patients, it was shown that DD was not a good marker of recurrence, while P-selectin was a good marker [29]. Subsequently, a systematic review on the predictive value of DD for recurrences in cancer patients that included 1433 patients showed a hazard ratio of 3,21 (95% CI, 1,25–8,25) [30]. In a pilot study conducted in our patient cohort [31], we quantified in a subset of CAT patients (N = 114) DD and hs-CRP at anticoagulation withdrawal and 21 days later. We found a significant increase in both markers in CAT patients who suffered a VTE recurrence compared to those who did not. In the present study, we extended the pilot study by evaluating additional coagulation-related parameters for a longer period to monitor the risk of VTE recurrences in CAT patients during 6 months after anticoagulation withdrawal. When we analyzed these markers in the entire cohort of CAT patients (N = 166) these differences remained significant. Herein, we recruited 166 cancer patients who experienced a VTE event and who were under anticoagulant treatment. We followed them for 6 months after anticoagulation withdrawal and quantified several coagulation-related parameters: DD, sP-selectin, hs-CRP, FVIII, TF, PPL activity and TGT at three time points (0, 21 and 90 days after anticoagulation withdrawal).

The rate of VTE recurrences in our cohort of CAT patients at 6 months of anticoagulation withdrawal was 9.6%, comparable to that obtained in previous studies [29,30]. Regarding the biomarkers studied, we found a significant increase in DD, sP-selectin, hs-CRP and FVIII in CAT patients with VTE recurrence, while TF and PPL activity did not change in the course of follow-up. Additionally, we found an increase in several parameters of the TGT (lagtime, start tail and time to peak).

P-selectin is a constitutively expressed cell-adhesion protein in platelets and in the endothelial cells, with a soluble form present in plasma [32]. sP-selectin has been previously proposed as biomarker for CAT [33]. DD is one of the products released during fibrin degradation that specifically originates from polymerized fibrin [34]. DD has been widely proposed as a VTE biomarker and is particularly useful for its high negative predictive value [35]. Both molecules, sP-selectin and DD, have been included in the Vienna risk model for predicting VTE in cancer patients, improving the risk prediction proposed by the Khorana score [12].

Circulating TF has previously been related to thrombosis [36] and tumor cells have been proven to release TF-bearing microparticles the exhibit procoagulant activity [37]. The CATCH trial described TF as a potential biomarker for recurrent VTE in cancer patients who were on anticoagulation treatment [38]. However, this biomarker may not be useful to predict VTE recurrences after anticoagulation withdrawal as, in our study, TF levels were similar during the follow-up of CAT patients after anticoagulation withdrawal.

Increased plasma levels of FVIII have been observed in certain malignancies and have been proposed as a CAT biomarker [39]. In line with these results, we evidenced a significant increase in FVIII levels 6 months after anticoagulant withdrawal in those CAT patients who experienced a VTE recurrence.

CRP is a plasma homopentameric acute-phase inflammatory protein that is released during inflammatory processes and can increase up to 1000-fold at sites of infection or inflammation [40]. CRP has previously been related to thrombosis and increased CRP levels have been independently associated with an increased risk of VTE [41,42] although with controversial results [43]. In our cohort of CAT patients, we observed a significant increase in hs-CRP levels 21 days and 90 days after anticoagulant withdrawal in patients who suffered a VTE recurrence. Unfortunately, we do not have data that allow us to answer precisely whether elevated CRP could be a causal link for VTE recurrence or could be an indicator of high-grade inflammation associated with cancer.

Regarding the TGT, Lundbech M. et al. [44] studied different biomarkers in order to assess the thrombotic risk in low-stage primary lung cancer patients and head and neck cancer patients. They observed a prolonged lag time, lower thrombin peak, prolonged time to peak and lower ETP in cancer patients compared to healthy individuals. In our cohort of patients, we found a similar effect with a prolonged lagtime, time to peak and starttail in those patients suffering VTE recurrence. This is certainly an unexpected outcome that, as suggested by Lundbech M. et al., may be caused by depletion in coagulation factors in the plasma assayed in the TGT caused by an increase in TG at the tumor site (an increased in vivo production of thrombin resulting in reduced potential to generate thrombin ex vivo, as an exhaustion phenomenon), as it has also been observed in other pathological situations [45,46].

This study has shown that the biomarkers 21 days after stopping anticoagulation, such as DD/baseline DD ratio (DD ratio) > 2, hs-CRP > 4.5 mg/L and sP-selectin > 40 ng/dL, could predict VTE recurrences in CAT patients with a high predictive negative value, outperforming that of the Ottawa score in our CAT cohort. These results are of great clinical interest since they are parameters of easy implementation.

This analysis would provide the basis for future larger studies to validate the results. To the best of our knowledge, this is the first study that describes a profile of markers to estimate the individual risk of VTE recurrences in CAT patients in a dynamic way, which may allow the identification of the optimal time to discontinue anticoagulation in each CAT patient.

Several limitations in our study ought to be considered. The restoration of anticoagulation in those CAT patients with a clinical suspicion of high VTE recurrence probability may have diminished the number of VTE recurrences observed along the follow-up period. However, the patient’s safety and survival were always our priority, thus this drawback cannot be circumvented in a study design such as ours. Regarding the studied biomarkers, clinicians must be aware that DD increase in many clinical conditions. Physiologic causes of DD increase include pregnancy and puerperium, increasing age (>65 years), African American heritage, cigarette smoking, recent trauma and the postoperative period [35]. Strengths of our study are the relatively large cohort of 166 CAT patients studied, with blood sampling at three different time points and a thorough clinical evaluation during a follow-up period of 6 months after anticoagulation withdrawal. Moreover, the wide variety of different tumors studied guarantees that our results are not biased for only one class of tumor.

## 5. Conclusions

The optimal duration of anticoagulant treatment in cancer patients remains a challenge. In this study we have identified that the ratio (21-day DD/baseline DD) > 2, hs-CRP > 4.5 mg/L and sP-selectin > 40 ng/dL, determined 21 days after the discontinuation of anticoagulation, are potential biomarkers of VTE recurrence in CAT patients. These parameters have shown high predictive negative values. A risk-adapted strategy could identify the optimal time to stop the anticoagulation in CAT patients in a tailored manner.

## Figures and Tables

**Table 1:** Clinical characteristics of the patients studied.

	Total N = 166	VTE Recurrence at 3 Months N = 8	VTE Recurrence at 6 Months N = 16
Sex male, N (%)	89 (53.6)	6 (75.0)	11 (68.8)
Age (y), median (IQR)	64 (17.0)	64.5 (23.5)	63.5 (16.0)
Weight (Kg), median (IQR)	71 (20.0)	82.0 (19.0)	79.5 (14.0)
Cancer location, N (%)			
Gastrointestinal	35 (21.1)	0 (0.0)	1 (6.2)
Breast and Ovarian	30 (18.1)	1 (12.5)	3 (18.8)
Hematologic	28 (16.9)	1 (12.5)	2 (12.5)
Urinary	26 (15.6)	1 (12.5)	3 (18.8)
Lung	19 (11.4)	3 (37.5)	5 (31.2)
Cerebral	3 (1.8)	1 (12.5)	1 (6.2)
Other	25 (15.1)	1 (12.5)	1 (6.2)
VTE, N (%)			
DVT	89 (53.6)	6 (75.0)	11 (68.8)
PE	47 (28.3)	0 (0.0)	0 (0.0)
DVT + PE	18 (10.8)	2 (25.0)	5 (31.2) *
Atypical VTE	12 (7.2)	0 (0.0)	0 (0.0) *
PT g.20210G>A, N (%)	6 (5.0)	0 (0.0)	1 (10.0)
FV Leiden, N (%)	10 (6.0)	2 (25.0)	2 (20.0)
Anti-phospholipid antibody, N (%)	2 (1.7)	0 (0.0)	0 (0.0)
Situation at anticoagulant therapy discontinuation, N (%)			
ECOG status ≥ 2	16 (9.6)	1 (12.5)	3 (18.8)
Active metastasis	65 (39.2)	5 (62.5)	9 (56.2)
Active cancer treatment Hormone therapy Alkylating agents Anti-metabolites Immune checkpoint inhibitors Monoclonal antibodies	56 (33.7) 20 (12.0) 3 (1.8) 15 (9.0) 5 (3.0) 8 (4.8)	4 (50.0) 2 (25.0) 1 (12.5) 0 (0.0) 2 (25.0) 0 (0.0)	9 (56.2) * 3 (18.8) 1 (6.3) 3 (18.8) 2 (12.5) 0 (0.0)
CV Catheter	12 (7.2)	1 (12.5)	1 (6.2)
Months of anticoagulant treatment, median (IQR)	10 (11.0)	18 (15.0)	12 (13.0)
DVT resolution, N (%)	72/107 (67.2)	5/8 (62.5)	9/16 (56.2)
PE resolution, N (%)	53/65 (81.5)	2/2 (100.0)	3/5 (60.0)

* *p* < 0.05; PT g.20210G>A, FV Leiden and anti-phospholipid antibody analyses were performed in 120 patients. VTE: venous thromboembolism; IQR: interquartile range; DVT: deep vein thrombosis; PE: pulmonary embolism; PT: prothrombin; CV: central venous. A patient could receive several drugs of different types of cancer treatment.

**Table 2:** Hazar ratio competing risk.

	HR	CI 95%	sHR	CI 95%	*p*
hs-CRP > 4.5 mg/L	5.10	1.27–20.39	4.77	1.26–18.07	0.021
Ratio (21-day DD/baseline DD) > 2	6.00	1.52–23.72	6.51	1.55–27.25	0.010
sP-selectin > 40 ng/dL	5.72	1.51–21.59	5.58	1.58–19.75	0.007
Male sex	3.58	0.89–14.36	1.29	0.86–15.33	0.079
Age (years)	1.00	0.96–1.05	1.01	0.99–1.04	0.760
Active metastasis	2.94	0.75–11.43	2.78	0.51–14.9	0.230
Active treatment	1.67	0.46–5.98	1.77	0.40–7.77	0.450

HR: hazard ratio of VTE recurrence without death competing risk; sHR: hazard ratio of recurrence adjusted with competing risk. Concordance = 0.890 (se = 0.041); Likelihood ratio test = 38.05 on 7 degrees of freedom, *p* = 3 × 10^−6;^ Wald test = 29.45 on 7 degrees of freedom, *p* = 1 × 10^−4;^ Score (logrank) test = 43.09 on 7 degrees of freedom, *p* = 3 × 10^−7^.

**Table 3:** Sensitivity, specificity and predictive values of the selected biomarkers to estimate VTE recurrences in CAT patients.

	Sen %	95% CI	Spec %	95% CI	PNV %	95% CI	PPV %	95% CI
DD ratio * > 2	62.5	35.9–83.7	65.3	57.1–72.8	94.2	87.4–97.6	16.3	8.4–28.1
sP-selectin > 40ng/dL	76.9	45.9–93.8	72.5	64.1–79.5	97.1	91.1–99.2	20.8	11.0–35.4
hs-CRP > 4.5mg/L	75.0	47.4–91.7	70.7	62.6–77.7	96.4	90.4–98.8	21.4	12.0–34.8
All (and)	43.7	20.7–69.4	98.0	93.8–99.5	94.2	89.0–97.2	70.0	35.7–91.9
All (or)	93.7	67.7–99.6	36.7	29.1–44.	98.2	89.2–99.9	13.6	8.1–21.8
Modified Ottawa score ≥ 1 (high risk)	22.7	10.31–43.4.1	84.0	77.2–89.1	87.7	81.2–92.2	17.9	7.9–35.6
Modified Ottawa score ≥ 1 (high risk)	38.9	31.3–47.0	63.6	43.0–80.3	13.7	8.4–21.7	36.4	19.7–57.0

* DD ratio: 21-day DD/baseline DD; All (and): all biomarkers exceed the cutoff value vs. any other result: three biomarkers below the cutoff negative or any of them below the cutoff; All (or): some biomarker(s) exceed the cutoff value; Sen: sensitivity; CI: confidence interval; Spec: specificity; PNV: predictive negative value; PPV: predictive positive value.

## Data Availability

The data presented in this study are available upon reasonable request to the corresponding author.

## Supplementary Materials

The following are available online at https://www.mdpi.com/article/10.3390/cancers14112771/s1, Table S1: Clinical characteristics of the patients treated with LMWH beyond 6 months.

## References

[1] Blom J.W., Doggen C.J.M., Osanto S., Rosendaal F.R. (2005). Malignancies, prothrombotic mutations, and the risk of venous thrombosis. JAMA.

[2] Sørensen H.T., Mellemkjær L., Olsen J.H., Baron J.A. (2000). Prognosis of cancers associated with venous thromboembolism. N. Engl. J. Med..

[3] Abdol Razak N.B., Jones G., Bhandari M., Berndt M.C., Metharom P. (2018). Cancer-associated thrombosis: An overview of mechanisms, risk factors, and treatment. Cancers.

[4] Paneesha S., McManus A., Arya R., Scriven N., Farren T., Nokes T., Bacon S., Nieland A., Cooper D., Smith H. (2010). Frequency, demographics and risk (according to tumour type or site) of cancer-associated thrombosis among patients seen at outpatient DVT clinics. Thromb. Haemost..

[5] Chew H.K., Wun T., Harvey D., Zhou H., White R.H. (2006). Incidence of Venous Thromboembolism and Its Effect on Survival among Patients With Common Cancers. Arch. Intern. Med..

[6] Chee C.E., Ashrani A.A., Marks R.S., Petterson T.M., Bailey K.R., Melton L.J., Heit J.A. (2014). Predictors of venous thromboembolism recurrence and bleeding among active cancer patients: A population-based cohort study. Blood J. Am. Soc. Hematol..

[7] Khorana A.A., Kuderer N.M., Culakova E., Lyman G.H., Francis C.W. (2008). Development and validation of a predictive model for chemotherapy-associated thrombosis. Blood.

[8] Patell R., Rybicki L., McCrae K.R., Khorana A.A. (2017). Predicting risk of venous thromboembolism in hospitalized cancer patients: Utility of a risk assessment tool. Am. J. Hematol..

[9] Kruger S., Haas M., Burkl C., Goehring P., Kleespies A., Roeder F., Gallmeier E., Ormanns S., Westphalen C.B., Heinemann V. (2017). Incidence, outcome and risk stratification tools for venous thromboembolism in advanced pancreatic cancer—A retrospective cohort study. Thromb. Res..

[10] Muñoz Martín A.J., Ortega I., Font C., Pachón V., Castellón V., Martínez-Marín V., Salgado M., Martínez E., Calzas J., Rupérez A. (2018). Multivariable clinical-genetic risk model for predicting venous thromboembolic events in patients with cancer. Br. J. Cancer.

[11] Fuentes H.E., Paz L.H., Wang Y., Oramas D.M., Simons C.R., Tafur A.J. (2018). Performance of Current Thromboembolism Risk Assessment Tools in Patients with Gastric Cancer and Validity After First Treatment. Clin. Appl. Thromb. Off. J. Int. Acad. Clin. Appl. Thromb..

[12] Ay C., Dunkler D., Marosi C., Chiriac A.-L., Vormittag R., Simanek R., Quehenberger P., Zielinski C., Pabinger I. (2010). Prediction of venous thromboembolism in cancer patients. Blood J. Am. Soc. Hematol..

[13] Tafur A.J., Caprini J.A., Cote L., Trujillo-Santos J., Del Toro J., Garcia-Bragado F., Tolosa C., Barillari G., Visona A., Monreal M. (2017). Predictors of active cancer thromboembolic outcomes. RIETE experience of the Khorana score in cancer-associated thrombosis. Thromb. Haemost..

[14] Van Es N., Di Nisio M., Cesarman G., Kleinjan A., Otten H.-M., Mahé I., Wilts I.T., Twint D.C., Porreca E., Arrieta O. (2017). Comparison of risk prediction scores for venous thromboembolism in cancer patients: A prospective cohort study. Haematologica.

[15] Metcalf R.L., Al-Hadithi E., Hopley N., Henry T., Hodgson C., McGurk A., Mansoor W., Hasan J. (2017). Characterisation and risk assessment of venous thromboembolism in gastrointestinal cancers. World J. Gastrointest. Oncol..

[16] Khorana A.A., Francis C.W. (2018). Risk prediction of cancer-associated thrombosis: Appraising the first decade and developing the future. Thromb. Res..

[17] Delluc A., Miranda S., den Exter P., Louzada M., Alatri A., Ahn S., Monreal M., Khorana A., Huisman M.V., Wells P.S. (2020). Accuracy of the Ottawa score in risk stratification of recurrent venous thromboembolism in patients with cancer-associated venous thromboembolism: A systematic review and meta-analysis. Haematologica.

[18] Douketis J., Tosetto A., Marcucci M., Baglin T., Cosmi B., Cushman M., Kyrle P., Poli D., Tait R.C., Iorio A. (2011). Risk of recurrence after venous thromboembolism in men and women: Patient level meta-analysis. BMJ Clin. Res. Ed..

[19] Lyman G.H., Khorana A.A., Falanga A., Clarke-Pearson D., Flowers C., Jahanzeb M., Kakkar A., Kuderer N.M., Levine M.N., Liebman H. (2007). American Society of Clinical Oncology guideline: Recommendations for venous thromboembolism prophylaxis and treatment in patients with cancer. J. Clin. Oncol. Off. J. Am. Soc. Clin. Oncol..

[20] Watson H.G., Keeling D.M., Laffan M., Tait R.C., Makris M., British Committee for Standards in Haematology (2015). Guideline on aspects of cancer-related venous thrombosis. Br. J. Haematol..

[21] Mandala M., Falanga A., Roila F. (2011). Management of venous thromboembolism (VTE) in cancer patients: ESMO Clinical Practice Guidelines. Ann. Oncol..

[22] Kearon C., Akl E.A., Ornelas J., Blaivas A., Jimenez D., Bounameaux H., Huisman M., King C.S., Morris T.A., Sood N. (2016). Antithrombotic therapy for VTE disease: CHEST guideline and expert panel report. Chest.

[23] Wojtukiewicz M.Z., Skalij P., Tokajuk P., Politynska B., Wojtukiewicz A.M., Tucker S.C., Honn K.V. (2020). Direct Oral Anticoagulants in Cancer Patients. Time for a Change in Paradigm. Cancers.

[24] Key N.S., Khorana A.A., Kuderer N.M., Bohlke K., Lee A.Y.Y., Arcelus J.I., Wong S.L., Balaban E.P., Flowers C.R., Francis C.W. (2020). Venous thromboembolism prophylaxis and treatment in patients with cancer: ASCO clinical practice guideline update. J. Clin. Oncol..

[25] Farge D., Frere C., Connors J.M., Ay C., Khorana A.A., Munoz A., Brenner B., Kakkar A., Rafii H., Solymoss S. (2019). 2019 international clinical practice guidelines for the treatment and prophylaxis of venous thromboembolism in patients with cancer. Lancet Oncol..

[26] Muñoz Martín A.J., Gallardo Díaz E., García Escobar I., Macías Montero R., Martínez-Marín V., Pachón Olmos V., Pérez Segura P., Quintanar Verdúguez T., Salgado Fernández M. (2020). SEOM clinical guideline of venous thromboembolism (VTE) and cancer (2019). Clin. Transl. Oncol. Off. Publ. Fed. Span. Oncol. Soc. Natl. Cancer Inst. Mex..

[27] Streiff M.B., Holmstrom B., Angelini D., Ashrani A., Elshoury A., Fanikos J., Fertrin K.Y., Fogerty A.E., Gao S., Goldhaber S.Z. (2021). Cancer-Associated Venous Thromboembolic Disease (Version 2.2021). J. Natl. Compr. Cancer Netw..

[28] Nishimoto Y., Yamashita Y., Morimoto T., Saga S., Amano H., Takase T., Hiramori S., Kim K., Oi M., Akao M. (2020). Predictive ability of modified Ottawa score for recurrence in patients with cancer-associated venous thromboembolism: From the COMMAND VTE Registry. Thromb. Res..

[29] Van Es N., Louzada M., Carrier M., Tagalakis V., Gross P.L., Shivakumar S., Rodger M.A., Wells P.S. (2018). Predicting the risk of recurrent venous thromboembolism in patients with cancer: A prospective cohort study. Thromb. Res..

[30] Yang M., Qi J., Tang Y., Wu D., Han Y. (2020). Increased D-dimer predicts the risk of cancer-associated recurrent venous thromboembolism and venous thromboembolism: A systematic review and meta-analysis. Thromb. Res..

[31] Jara-Palomares L., Solier-Lopez A., Elias-Hernandez T., Asensio-Cruz M.I., Blasco-Esquivias I., Sanchez-Lopez V., de la Borbolla M.R., Arellano-Orden E., Suarez-Valdivia L., Marin-Romero S. (2018). D-dimer and high-sensitivity C-reactive protein levels to predict venous thromboembolism recurrence after discontinuation of anticoagulation for cancer-associated thrombosis. Br. J. Cancer.

[32] André P. (2004). P-selectin in haemostasis. Br. J. Haematol..

[33] Ay C., Simanek R., Vormittag R., Dunkler D., Alguel G., Koder S., Kornek G., Marosi C., Wagner O., Zielinski C. (2008). High plasma levels of soluble P-selectin are predictive of venous thromboembolism in cancer patients: Results from the Vienna Cancer and Thrombosis Study (CATS). Blood J. Am. Soc. Hematol..

[34] Linkins L., Takach Lapner S. (2017). Review of D-dimer testing: Good, Bad, and Ugly. Int. J. Lab. Hematol..

[35] Pulivarthi S., Gurram M.K. (2014). Effectiveness of d-dimer as a screening test for venous thromboembolism: An update. N. Am. J. Med. Sci..

[36] Rauch U., Nemerson Y. (2000). Circulating tissue factor and thrombosis. Curr. Opin. Hematol..

[37] Davila M., Amirkhosravi A., Coll E., Desai H., Robles L., Colon J., Baker C.H., Francis J.L. (2008). Tissue factor-bearing microparticles derived from tumor cells: Impact on coagulation activation. J. Thromb. Haemost..

[38] Khorana A.A., Kamphuisen P.W., Meyer G., Bauersachs R., Janas M.S., Jarner M.F., Lee A.Y.Y. (2017). Tissue factor as a predictor of recurrent venous thromboembolism in malignancy: Biomarker analyses of the CATCH trial. J. Clin. Oncol..

[39] Vormittag R., Simanek R., Ay C., Dunkler D., Quehenberger P., Marosi C., Zielinski C., Pabinger I. (2009). High factor VIII levels independently predict venous thromboembolism in cancer patients: The cancer and thrombosis study. Arterioscler. Thromb. Vasc. Biol..

[40] Sproston N.R., Ashworth J.J. (2018). Role of C-reactive protein at sites of inflammation and infection. Front. Immunol..

[41] Folsom A.R., Lutsey P.L., Astor B.C., Cushman M. (2009). C-reactive protein and venous thromboembolism. Thromb. Haemost..

[42] Grimnes G., Isaksen T., Tichelaar Y.I.G.V., Brox J., Brækkan S.K., Hansen J.-B. (2018). C-reactive protein and risk of venous thromboembolism: Results from a population-based case-crossover study. Haematologica.

[43] Vormittag R., Vukovich T., Schönauer V., Lehr S., Minar E., Bialonczyk C., Hirschl M., Pabinger I. (2005). Basal high-sensitivity-C-reactive protein levels in patients with spontaneous venous thromboembolism. Thromb. Haemost..

[44] Lundbech M., Krag A.E., Christensen T.D., Hvas A.-M. (2020). Thrombin generation, thrombin-antithrombin complex, and prothrombin fragment F1+ 2 as biomarkers for hypercoagulability in cancer patients. Thromb. Res..

[45] Hansen C.H., Ritschel V., Halvorsen S., Andersen G.Ø., Bjørnerheim R., Eritsland J., Arnesen H., Seljeflot I. (2015). Markers of thrombin generation are associated with myocardial necrosis and left ventricular impairment in patients with ST-elevation myocardial infarction. Thromb. J..

[46] Bratseth V., Pettersen A.-Å., Opstad T.B., Arnesen H., Seljeflot I. (2012). Markers of hypercoagulability in CAD patients. Effects of single aspirin and clopidogrel treatment. Thromb. J..

