# Supplementary material for: Biomarkers of Venous Thromboembolism Recurrence after Discontinuation of Low Molecular Weight Heparin Treatment for Cancer-Associated Thrombosis (HISPALIS-Study)
Source: Cancers (Basel). 2022 Jun 2;14(11):2771. doi: 10.3390/cancers14112771 (PMC9179374; doi:10.3390/cancers14112771)
Supplement: Supplementary file 1 [file cancers-14-02771-s001.zip › cancers-1741907-supplementary.pdf]

cancers

MDPI

Supplementary Material

Biomarkers of Venous Thromboembolism Recurrence After Discontinuation of Low Molecular Weight Heparin Treatment for Cancer-Associated Thrombosis (HISPALIS-Study)

Remedios Otero, Aurora Solier-López, Verónica Sánchez-López, Julia Oto, Elena Arellano, Samira Marín, Luis Jara-Palomares, Teresa Elías, María Isabel Asencio, Isabel Blasco-Esquivias, María Rodríguez de la Borbolla, José María Sánchez-Díaz, Macarena Real-Domínguez, Emilio García-Cabrera, Francisco Javier Rodríguez-Martorell, and Pilar Medina

Table S1. Clinical Characteristics of the patients treated with LMWH beyond 6 months.

	Stopped anticoagulant therapy (Included patients) N = 166	Not stopped anticoagulant therapy (Excluded patients) N = 211
**Sex male, N (%)**	89 (53.6)	121 (57.4)
**Age (y), median (IQR)**	64 (17)	63.6 (16)
**Weight (Kg), median (IQR)**	71 (20)	75.0 (20)
**Cancer location, N (%)**		
**Cerebral**	3 (1.8)	5 (2.4)
**Digestive**	35 (21.1)	59 (28)
**Hematologic**	28 (16.9)	13 (6)
**Breast and Ovarian**	30 (18.1)	33 (15.5)
**Lung**	19 (11.4)	40 (19)
**Urinary**	26 (15.6)	33 (15.5)
**Other**	25 (15.1)	28 (13)
**VTE, N (%)**		
**DVT**	89 (53.6)	113 (53.4)
**PE**	47 (28.3)	51(24)
**DTV + PE**	18 (10.8)	33 (15.5)
**Atypical VTE**	12 (7.2)	14 (6.8)
**Incidental VTE**	50 (30)	89 (42.2)
**PT g. 20210G>A, N (%)**	6 (5.0)	5 (11.0)
**FV Leiden, N (%)**	10 (6.0)	5 (11.0)
**Anti-phospholipid antibody, N (%)**	2 (1.7)	1 (2.2)
**ECOG status ≥ 2**	16 (9.6)	38 (18.1)
**Active metastasis, N (%)**	65 (39.2)	147 (70)
**Chemotherapy, N (%)**	56 (33.7)	125 (59.4)
**CV Catheter, N (%)**	12 (7.2)	29 (13.7)
**Months of anticoagulant treatment, median (IQR)**	11 (11)	38 (18)
**DVT resolution, N (%)**	72/107 (67.2)	45/145 (31)
**PE resolution, N (%)**	53/65 (81.5)	58/84 (69)

Prothrombin gene mutation g.20210G>A, FV Leiden and anti-phospholipid antibody analysis was performed in 120 patients in the stopped anticoagulant therapy group and in 45 patients in the not stopped anticoagulant therapy group. LMWH: low-molecular-weight heparin; IQR: interquartile range; VTE: venous thromboembolism; DVT: deep vein thrombosis; PE: pulmonary embolism; PT: prothrombin; CV: central venous.
